# Prevention of pressure ulcers, malnutrition, poor oral health and falls in nursing homes: A focus group study with nurse aides, registered nurses and managers

**DOI:** 10.1016/j.ijnsa.2021.100056

**Published:** 2021-11-30

**Authors:** Merita Neziraj, Magdalena Andersson, Peter Hellman, Malin Axelsson, Christine Kumlien

**Affiliations:** a Department of Care Science, Faculty of Health and Society, Malmö University, Sweden; b Department of Health and Social Care, Strategic Development, Unit of Research, Quality and Education, Malmö, Sweden; c Department of Cardio-Thoracic and Vascular Surgery, Skane University Hospital, Malmö, Sweden

**Keywords:** Elderly, Falls, Focus group, Health care staff, Malnutrition, Managers, Poor oral health, Pressure ulcers, Prevention, Qualitative

## Abstract

**Background:** Despite available knowledge how to prevent the risk of pressure ulcers, malnutrition, poor oral health and falls among older persons in nursing homes, these risks still frequently occur and cause a major burden for older persons; furthermore, for the health care system, they are extremely costly. One way to combat these risks is to register the prevention process in quality registries. However, the increasing older population worldwide is going to put high demands on those working with this group of people.

**Objective:** To explore how nurse aides, registered nurses and managers in nursing homes experience working with the prevention of pressure ulcers, malnutrition, poor oral health and falls in general and according to the quality register Senior Alert care process.

**Methods:** A qualitative study was conducted in nursing homes in a municipality in southern Sweden. We purposively sampled nurse aides, registered nurses and managers (*n* = 21) working in nursing homes registered in the quality register Senior Alert, who then participated in one of five focus group semistructured digital interviews held between February and April 2020. The interviews were audio recorded. Data were analysed using reflexive thematic analysis.

**Results:** Our findings explore how nurse aides, registered nurses and managers experience working with the prevention of pressure ulcers, malnutrition, poor oral health and falls in nursing homes both in general and according to Senior Alert. The following four themes were generated during the analysis: (1) is included in the everyday work, (2) requires team effort, (3) requires handling many challenges and (4) requires finding strategies.

**Conclusion:** The prevention of pressure ulcers, malnutrition, poor oral health and falls among older persons in nursing homes is complex. There is a commitment and responsibility among nurse aides, registered nurses and managers regarding preventive work and team effort, and finding useful strategies is necessary for the work to be successful. However, challenges, both at the individual and organizational levels, are involved, which implies that smoother organizational routines facilitating this preventive work are needed. Although nurse aides, registered nurses and managers are good at finding strategies that facilitate this work, one of the main challenges seems to lie in the variety of knowledge found among those working in nursing homes, particularly among nurse aides. This challenge was voiced by all the professionals, which suggests the need for a tailored educational intervention aimed at increasing the related knowledge among those working in nursing homes to enhance preventive work.

**What is already known?**

• The prevalence of the risk for pressure ulcers, malnutrition, poor oral health and falls is high, which implies that these health risks are a great concern for older persons, thereby causing a major burden for them and raising high for the entire health care system.
• An increasing aging population is going to put high demands on health care staff and managers working with older persons in nursing homes.

**What this paper adds?**

• There is a commitment and responsibility regarding preventive work found among nurse aides, registered nurses and managers in nursing homes.
• Preventive work in nursing homes has to be done in a complex environment, since it is a part of everyday work, requires team effort, and requires handling individual and organizational challenges, as well as finding strategies that facilitate this work.
• To prevent these health risks, an increased level of knowledge, particularly among nurse aides, and smoother organizational routines facilitating this preventive work are warranted.

## Introduction

The risk for the occurrence of pressure ulcers, malnutrition, poor oral health and falls among older persons in nursing homes is frequent (Neziraj et al., 2021; Trinks et al., 2018), which implies that these health risks are a great concern for older persons receiving municipal health care. Because the number of older persons will double by 2050 (United Nations, 2017), and the largest increase is expected in the 80-years-and-older age group (WHO, 2014), health care staff and managers in nursing homes in particular are expected to face the related health demands that follow, including an increased risk for pressure ulcers, malnutrition, poor oral health and falls among older persons (Neziraj et al., 2021). Therefore, the importance of preventing these health risks among older persons cannot be overemphasized. Alarmingly, despite the existing evidence-based knowledge about how to prevent these health risks, the prevalence of risk for these health risks is still high among older persons in nursing homes (Neziraj et al., 2021; Trinks et al., 2018), which causes a major burden for older persons and has a negative impact on their health (Gorecki et al., 2009). For the health care system, they are extremely costly (Marjolein van der et al., 2020). A lack of knowledge among those working in nursing homes (Dalvand et al., 2018) and organizational factors (Lannering et al., 2017a, 2017b), for instance, impedes this work. It is therefore warranted to explore how health care staff and managers experience preventive work regarding these health risks. Knowledge from the current study can increase the understanding of different professionals’ perspectives regarding the prevention of these health risks and, importantly, increase the possibility of improvement in this area.

## Background

Older persons are more vulnerable to negative outcomes, such as the risk for pressure ulcers, malnutrition, poor oral health and falls (Bravell et al., 2011). The causes of these health risks are multifactorial, and they seldom occur in isolation (Lannering et al., 2016). To enable healthy aging in older persons, the prevention of these health risks is central to good quality of care. However, these health risks continue to be worldwide problems in health care (Bååth et al., 2014). In Sweden, approximately 30% (Neziraj et al., 2021) and internationally, 4–31% (Kottner et al., 2010; VanDenKerkhof et al., 2011) of older persons in nursing homes are at risk for pressure ulcers. Regarding the risk for malnutrition, nationally, 40–55% (Borgström Bolmsjö et al., 2015; Neziraj et al., 2021) and internationally, 15–40% (Verbrugghe et al., 2013) of older persons in nursing homes are at risk. It is well underpinned that pressure ulcers are associated with malnutrition, suggesting the importance of preventing the risk for malnutrition in the presence of the risk for pressure ulcers.

Among malnourished older persons, there is an increased risk for poor oral health (Van Lancker et al., 2012) and an increased risk for falls (Westergren et al., 2014), which indicates that malnutrition, poor oral health and falls are interrelated and thus emphasizes the importance of preventing all these health risks among older persons. In Sweden, 42% are at risk for poor oral health (Bellander et al., 2021), and internationally, oral health problems are common among older persons in nursing homes (Thomson DR, 2014). Poor oral health is a serious health and economic burden that reduces the quality of life for those affected (Peres et al., 2019), which suggests that oral health should be included as a natural part of nursing work.

Because falls are the second leading cause of accidental injury deaths worldwide (WHO, 2021) and in Sweden, 90% of all injuries among older persons 80 years and older are caused by a fall (National Board of Health and Welfare, 2018), preventing falls is crucial. The risk for falls is, in turn, associated with the risk for malnutrition (Lannering et al., 2017a, 2017b), thereby stressing the need for comprehensive preventive work regarding all the above-mentioned health risks among older persons in nursing homes. These health risks can lead to an increased risk of morbidity, mortality, hospitalization and nursing home admissions among older persons (Laurence, 2006, Lannering et al., 2017b). Consequently, preventing these health risks is both a human and a societal priority.

In Sweden, there is a national web-based quality registry, namely, Senior Alert, which supports an individualized, standardized, structured and systematic preventive work process for older persons 65 years or older at risk for pressure ulcers, malnutrition, poor oral health and falls. Senior Alert provides risk assessment using different instruments, analyses the causes when risks occur, planning and performing care interventions, and evaluates the interventions (Edvinsson et al., 2015), which are often conducted by registered nurses and nurse aides. Using a preventive process, similar to the one outlined by Senior Alert, decreases the risk of morbidity and mortality among older persons and can increase cost efficiency (Rubenstein et al., 2001). However, despite the availability of such methods to prevent these health risks, not all older persons who are at risk have planned care interventions (Gusdal et al., 2021; Backlund et al., 2018); furthermore, there is often a mismatch between risk and planned care interventions (Witt et al., 2018). Additionally, the lack of specialist registered nurses in the field of elderly care in Sweden and barriers in the organizational structures such as managers not understanding the preventive care process and thus not prompting a good learning process impede this work (Lannering et al., 2017a, 2017b). Such impediments pose a serious risk for patient safety for older persons (National Board of Health and Welfare, 2021), which suggests and yells for improvement in this regard.

Considering the existing problems with the prevalence of the health risks among older persons, the difficulties related to health care staff and managers preventing them and thus meeting the demographic challenges of the aging population, it is warranted for improvement in this area. To our knowledge, no previous study has explored nurse aides, registered nurses’ and managers’ experiences working with the prevention of pressure ulcers, malnutrition, poor oral health and falls either in general or according to the Senior Alert care process. The findings from the current study can increase the understanding of different professionals’ experiences regarding the prevention of these health risks and help us to better understanding why so many older persons still suffer from these risks. This can, in turn, support the development of a future intervention aiming to increase knowledge among those working in nursing homes regarding preventive work and thereby reduce the risk for pressure ulcers, malnutrition, poor oral health and falls among older persons in Swedish nursing homes.

## Aim

The aim of the current study was to explore how nurse aides, registered nurses and managers in nursing homes experience working with prevention of pressure ulcers, malnutrition, poor oral health and falls both in general and according to the quality register Senior Alert care process.

## Methods

The current qualitative study was conducted within the research project PROSENIOR (https://mau.se/en/research/projects/PROSENIOR-Risk-assessments-prevention-quality-registry-SeniorAlert/). The reporting of this study follows the consolidated criteria for reporting qualitative research (COREQ) (Tong et al., 2007).

### 4.1 Definitions

*Nurse aide (NA):* a person with a secondary degree in nursing, which involves three years of study at high school, *or* a person without any formal education in nursing.

*Registered nurse (RN):* a person with a bachelor's degree in nursing, which involves three years of study at university.

*Manager:* a person who is in charge of NAs *or* RNs.

### 4.2 Context

This study took place in public nursing homes in a municipality in southern Sweden. In Sweden, the care of older persons living in nursing homes is primarily a public responsibility and financed through taxes. Swedish health care is divided into three levels: national, regional and municipal. Municipalities are responsible for the care and services of older persons living in nursing homes. Care and services in Swedish nursing homes are provided within the Health and Social Services Act (HSL, 2017) and the Social Service Act (SFS, 2001). NAs work under the Social Service Act but are delegated tasks within the Health and Social Services Act, usually by RNs. NAs are on duty around the clock and are the main providers of care and services in nursing homes, while RNs guide care in nursing homes and work under the Health and Social Services Act. An RN (sometimes two, depending on the size of the nursing home) is accessible during office hours. Managers in charge of the care and services provided by the NAs are located at their respective nursing homes. Managers in charge of RNs are in charge of several RNs working at different nursing homes. Most often, older persons living in nursing homes are allocated to a physician who is employed by the county council and works at a primary health care center usually located near the nursing home. Physiotherapists and occupational therapists are situated outside the nursing homes and are referred to as the rehab team in this study. The rehab team focuses mainly on self-care and mobility. All health care and services provided in the nursing homes require recording by legislation.

### 4.3 Sample and recruitment process

The inclusion criteria were NAs, RNs and managers working in nursing homes registered in the quality registry named Senior Alert. A purposive sampling technique was used to select groups of individuals who contributed to answering our research question. In the first step of recruitment, the authors met with the head of nursing homes to discuss eligible nursing homes for the current study. Then, the first author informed the managers in charge of the eligible nursing homes about the study at digital workplace meetings. Each manager, in turn, informed the RNs and NAs about the study. In addition, RNs were further informed by the first author at digital workplace meetings, while NAs were further informed via a 5-minute long recorded film containing information about the current study on their workplace meetings. Written information about the study was distributed to NAs, RNs and managers working in eligible nursing homes.

### 4.4 Participants

In total, 9 nursing homes were eligible to participate in the study. NAs, RNs and managers working in these nursing homes who were interested in participating in the current study were encouraged to contact the first author to schedule time for interviews. Twenty-three participants agreed to participate at first, but 21 ultimately participated. One of the participants canceled due to acute tasks at work, and the other canceled due to illness.

### 4.5 Data collection

In total, five focus group interviews were conducted, two with NAs, two with RNs and one with managers. Homogenous focus group interviews were chosen to enhance the discussions within the same type of professionals. Although the participants individually answered the questions, the discussions encouraged the participants to interact and share their experiences. A semi-structured interviews guide was used. The focus groups consisted of 3–6 participants (Krueger and Casey, 2015). The first author, MN, facilitated as moderator, and either MA or MAX acted as a co-moderator. The role of the moderator was to open up the discussion with a broad question about the topic of interest before asking focused and probing questions (Table 1). The co-moderators asked complementary questions and took notes. Due to the current pandemic, all the focus group interviews took place digitally via Microsoft Teams, were the audio was recorded and lasted between 63 and 106 min (mean 83 min). Data were collected between February and April 2021. Demographic information was collected by individual questionnaires prior to the focus group interviews. The characteristics of the participants are presented in Table 2.

**Table 1:** Examples of questions in the focus group discussions.

**Broad question**	What do you think about pressure, ulcers, malnutrition, poor oral health and falls?
**Focused questions**	How do you work with risk assessments regarding pressure ulcers, malnutrition, poor oral health and fall? How do you work with preventive interventions regarding pressure ulcers, malnutrition, poor oral health and fall? Can you tell me about your experiences of registering risk assessments and interventions in Senior Alert?
**Probing questions**	Can you tell me more about that? Why do you think that is? In what why is that so?

**Table 2:** Characteristics of the participants (*n* = 21).

	Nurse aides	Registered nurses	Managers
Gender, n (female/male)	8/2	7/0	3/1
Age, mean (range)	50 (31–63)	46 (32–56)	48 (37–63)
Clinical experience in years, mean (range)	21 (5–43)	17 (6–31)	9 (4–16)
Education level, n			
Secondary degree	10*		1***
Bachelor's degree		4	3****
Master's degree		3**	

*All the nurse aides had a secondary degree diploma within nursing and an additional formal education considering the preventive process, according to quality registry Senior Alert; they were also responsible for the registration of risk assessments and care intervention in the registry. **Among the three specialist registered nurses; specialist nursing program, elderly care =1, specialist nursing program, primary health care =2. *** One of the managers had an additional advanced higher vocational education diploma within social psychiatry-dementia care and leadership. **** Among the three managers; study program in nursing =2, study program in social work =1.

### 4.6 Data analysis

The interviews were transcribed verbatim. Reflexive thematic analysis was chosen to identify patterns of meaning within the dataset (Braun and Clarke, 2006, 2019). Rather than starting with a specific theory, patterns of meaning were generated inductively, which means that coding and themes were generated by the data content. Furthermore, to reflect the explicit content of the data, themes were generated at a semantic level (Braun and Clarke, 2006, 2019). While the six-phase process according to Braun and Clarke (2019) is organized and provides research with core skills to conduct the analysis, the analytic process in this study was not a linear process moving forwards through these phases. Instead, this process required moving back and forth through the phases in a flexible manner to fit the data and the research question.

**Fig. 1:**
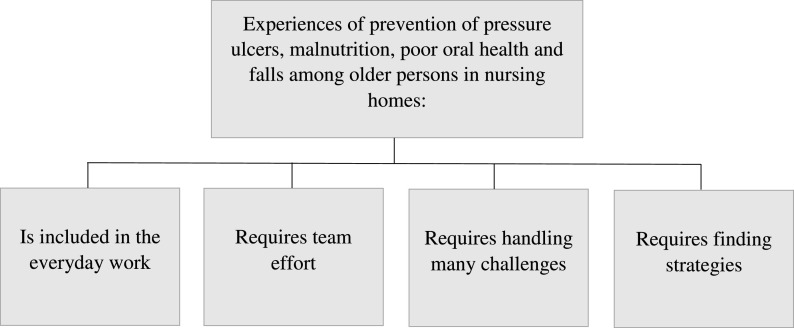
The four themes exploring how nurse aides, registered nurses and managers in nursing homes, experienced working with prevention of pressure ulcers, malnutrition, poor oral health and falls in general and according to the quality register Senior Alert care process.

First, all the authors read the transcripts. Then, each transcript was reread carefully by MN and CK. At this stage, initial ideas for coding were written down (familiarizing with the data). Second, MN and CK individually identified and coded entities of interest in the entire dataset (coding). In the third phase, mind maps were used to sort the initial codes into potential themes, and their meanings were discussed in relation to the research question by MN and CK (generating initial themes). In the fourth phase, the identified themes were either split or combined by MN and CK. Then, the themes were reviewed by all the authors to ensure that they worked in relation to the entire dataset (reviewing the identified themes). The fifth phase involved defining the themes by determining the story of each, before naming them, and finally reporting the findings (defining and naming the themes *and* producing the report). The analysis generated 4 themes, which are visualized in Fig. 1.

### 4.7 Rigor

In qualitative analysis, rigor belongs to the research process and its trustworthiness, the concepts of credibility, transferability and dependability (Lincoln and Guba, 1985). In an effort to obtain credibility, all the authors were involved in the analysis. In addition, an illustration of the analytic process is presented in Table 3, and quotations are reported in the results section below. The context and the participants are thoroughly described to achieve transferability. By making the research process transparent, dependability is supported. For instance, methodological decisions that were made are clearly articulated. For transparency, the authors’ positions are reported (Tong et al., 2007); four of the researchers hold positions as either doctoral students (MNs), senior lectures (PHs), associated professors (MAXs) or professors (CKs) at the affiliated university. MA is a PhD and works as a research and development coordinator in the municipality where the study was conducted. All the authors are registered nurses, and two of them (MN, MA) specialize in elderly care and have worked in this context previously. Preunderstanding cannot, therefore, be excluded; rather, it was identified, and the authors self-reflected and discussed it. For reflexivity, the first author (MN) wrote down thoughts during the whole process.

**Table 3:** Examples of the analytic process.

Data extract	Coding	Initial themes	Final themes
It is that pressure ulcers, if you are malnourished, it is easy to get pressure ulcers as well. In addition, they stick together… and then if oral health is not good either, you cannot eat. So, they are all related, all of them… they follow after each other. So, if you are observant, what see they have… their mouth first for them to eat properly, if they have good oral hygiene and everything possible in that regard so that they can eat properly. In addition, when they eat well, they also avoid pressure ulcers, I think.	The risks are related The risks affect each other Need to be observant of the risks Need to assess the risks By avoiding one risk, other risks can be avoided	A part of the daily work	Is included in the everyday work
/… We also have a physiotherapist and occupational therapist here and then the manager in the nursing home too. We all sit together with the nurse aides, preferably a nurse aide that knows the older person well, and then you sit and go through the care interventions. There may be something more you can do or there maybe something that has not worked as well so you can remove it.	Different professionals needed in the team Discussing and deciding care interventions together	The team is important	Requires team effort
Then, we can start talking about the competence of the nursing staff in order to lay a foundation for these risks to even be properly assessed by our nursing staff.	Lack of competence among nurse aides	Challenges	Requires handling many challenges
And then we have a whiteboard on each floor, in the staff room, where we write down what fall risks each person has, F, and O for oral health and M for malnutrition, so that all the substitutes and anyone can immediately see, and you can see when to do the next risk assessment, to make it clear…//… so that you see the whole picture of each older person in the everyday work…//… so you try make tools as clear as possible.	Whiteboards to make the preventive work clearer for staff Finds practical solutions	Local implementation strategies	Requires finding strategies

### 4.8 Ethical considerations

This study was conducted in accordance with the Declaration of Helsinki (World Medical Association, 2013) and approved by the Swedish Ethical Review Authority (Dnr 2019-06414). All the participants provided written informed consent before the focus group interviews were conducted.

## Results

The analysis of the discussions generated 4 themes (Fig. 1). Each theme is presented with an overview of the similarities among the professionals in all the focus groups, followed by an overview of the variations among the professionals. fg = focus group

### Is included in the everyday work

In all the discussions, the prevention of pressure ulcers, malnutrition, poor oral health and falls was experienced as being included in the everyday work. Furthermore, the participants reported experiencing that these health risks were all related and common among frail older persons.

From the perspective of the NAs, they described that they were especially skilled at preventing pressure ulcers. General care interventions were undertaken on a regular basis. For instance, the NAs described that they measured weight monthly. Individual care interventions, however, were planned and undertaken in accordance with the RN's recommendations. The NAs experienced that they observed the older persons on a daily basis and reported changes to the RN. One NA described it as follows: *And if someone does not eat during the week, then we take an extra weight on them and we report directly to the nurse…/(fg 4).*

The RNs shared the experience that the prevention of health risks was an important part of their work and responsibility. However, they experienced that some of the health risks could not be prevented at all times for different reasons. An RN explained it as follows: *Similar to malnutrition, but there can be some medical elements which maybe makes it so that you can't get around it or it is a long, long progressed dementia disease where you have all the swallowing difficulties and where for ethical reasons no tube is put in. Then, maybe you also get to that stage finally, but otherwise, I think it should not exist to the extent that it does (fg 3).* Furthermore, if the preventive work is done correctly from the beginning, this facilitates future work. Preventing health risks was described as enhancing the quality of care.

The managers discussed that health risks were common among older persons in nursing homes, and therefore, these health risks needed to be observed regularly. This was described by one of the managers as follows: *Of course I agree with the others in what they said, and when I think of these, oral health, falls and pressure ulcers, malnutrition, I immediately think that the older persons that we work towards almost everyone has problems with some of these. These are risks that exist when you get older that we need to be aware of and that we need to work actively with (fg 1).*

### Requires team effort

All the participants discussed that preventive work requires team effort. The participants experienced that the RN plays a crucial role on the team but that the NA knew the older person best; hence, both professionals were needed on the team. Furthermore, they described that admins (NAs with a formal education considering prevention, according to Senior Alert, and who are responsible for registration in the register) are key persons on the team for registering in Senior Alert. The role of the rehab team was described as important in preventing these health risks, especially the risk of falls. For the risk of poor oral health, a mobile home dental care team was related as being important.

From the perspective of the NAs, communication and collaboration with colleagues enhanced teamwork, which was described as follows: *Some (older persons) are slightly passive, so everyone makes an effort for them to move so that they don't get these pressure ulcers, and everything is about working together, that we work together and that we are observant, I think (fg 4).* Collaboration with the RN was also experienced as necessary in the preventive work, and a supportive RN facilitated this work. However, the NAs experienced that the RN was not required to be present all the time but was rather available when needed. Furthermore, the NAs described that a supportive manager enhanced their teamwork to prevent health risks.

The RNs described their own role in the team as very important. Communication and coherence within the team were described as necessary in the preventive work. Furthermore, the RNs pointed out that it was important to utilize all the available resources because different professions were experts within different areas. In their experience, relatives were sometimes also included on the team, and some mentioned that physicians should be included on the team. An engaging manager, however, was experienced as facilitating preventive work. One RN described it as follows: *Like you mentioned before X, this with not just sitting in the room or lying on the bed (the older persons). So much is probably about the mangers ‘commitment as well as the finical part, I would think (fg 2).*

The managers discussed that all the professions had an important role in the team. In their experience, RNs were crucial in regard to preventive work. They also highlighted the importance of the admin role, particularly in the registration part but also in educating their own colleagues in regard to everyday work. One manager explained as follows: *I would say that what works here is that we actually have very skilled, trained admins who know the system and can teach their colleagues…/(fg 1).*

### Requires handling many challenges

The participants discussed that preventive work in nursing homes requires handling many challenges. However, the professionals reported experiencing challenges at different levels. The NAs reported experiencing challenges related to older persons. The RNs and managers, on the other hand, discussed organizational factors as challenging. For instance, because RNs and NAs documented their notes in different record systems, their everyday work was impeded. Another challenge experienced by the RNs and managers was that the nursing record system and the Senior Alert register were not compatible, which led to double documentation. In all the interviews, the participants discussed that the lack of knowledge, particularly among NAs, hindered preventive work and was therefore experienced as a challenge.

From the perspective of the NAs, the risk assessment of oral health and planning and executing care interventions related to malnutrition among older persons with dementia diagnosis was experienced as being especially difficult: *Therefore, just like X is saying when working with older persons with dementia, they may not be able to open their mouth or understand, they may have come so far in their illness that they don't want to eat and such. So, you have to try with new things, so it might be a little harder there…/(fg 5).* Consequently, the NAs had to compromise with the older persons about what care interventions to undertake. The lack of communication and compliance among colleagues, as well as lack of time, were experienced as other challenges in regard to preventive work.

The RNs discussed that they would like to be involved in the everyday work, but instead they were leading the care. The RNs pointed out that basic patient care was usually done by the NAs; hence, the RNs had to rely on NAs assessments and reports. However, due to the lack of knowledge among the NAs regarding preventive work, the RNs reported experiencing that they constantly needed to repeat the basic knowledge. As a result, it was difficult to build up knowledge among them. One RN described it as follows: *No, it is exactly like that, because otherwise you could have built on and developed it as well, but it is just like starting over all the time (fg 2).* The unclear division of labor between the RNs and the managers who were responsible for actually driving the preventive work and educating the NAs was experienced as another challenge by the RNs. Furthermore, the Senior Alert itself was experienced as quite easy by the RNs, but its content was advanced, and they were unsure if the NAs fully understood the terminology. Therefore, there was a risk of incorrect registrations performed by the admins. Even if the NAs needed increased their knowledge in this regard, the RNs experienced that the NAs did not get opportunities for improvement in the same way as they did; this was expressed as follows by one RN: *However, that was it…we got it, that's the problem, that it stays with us. Because we were invited to this, and we were all like…this is great, and we brought home material and so on, but the nurse aides never go away on anything like this, that's the running point (fg 3).*

The managers discussed that if the NAs did not realize the benefits of working according to Senior Alert, it was difficult to involve them in everyday work. One manager described it as follows: *I just wanted to pick up a little bit of this, what X started with, what X continued with, to see a point in working with this system. Because I think that there is a lot that you need to work with to get this work together… So, therefore, I think that the nurse aides also do not truly understand the point of sitting and doing all this administrative work that it actually becomes for them. In addition, just this shows the benefits of what happens when you do the risk assessments…/(fg 1).* Moreover, the managers described several organizational challenges. First, if the mandatory record login cards were delayed, health care staff would not even have access to the record systems or Senior Alert. Second, the nursing record system in itself was not particularly user friendly, which meant that RNs spent a great amount of time documenting. High health care staff turnover was experienced as another organizational problem. The lack of mutual routines regarding the feedback process of preventive work, particularly to NAs, was experienced as another organizational challenge. To give the NAs some feedback, the process needs to be simplified, as explained by one of the managers: *…/I know it's difficult for them (nurse aides) to keep up if you show them graphs and pie charts and try to show them; this seems to have been good, and here we need to work better. I think you need to simplify the working method to be able to clearly show quickly and efficiently, when we have done this, this has happened (fg 1).*

### Requires finding strategies

In all the interviews, the participants discussed that preventive work requires finding strategies. Senior Alert was experienced as a working tool that facilitates everyday work by providing a preventive structured work process to reduce health risks among older persons. However, different strategies to implement the preventive work outlined by Senior Alert were described. For instance, in some nursing homes, they reported using whiteboards to obtain an overview of the health risks and what care interventions to undertake. In other nursing homes, risk assessments together with the planned care interventions were placed in folders situated in the older persons’ own apartments and thus became visible for all health care staff members. Undertaking general care interventions for all the older persons living in the nursing homes was described as another strategy. Structured meeting forms were another strategy that enhanced the entire preventive process but were experienced as explicitly necessary in the follow-up process.

From the perspective of the NAs, Senior Alert was experienced as helpful for obtaining an overview of the entire preventive process and to notice changes quickly. A NA explained: *…/I think it is a good tool to check older persons; you have a tool, and you can quickly check if, for example, that person has lost weight or they have fallen many times or have pressure ulcers as noted quite quickly… all our colleagues can see, if a person has lost weight or has fallen, if they (colleagues) were not present when it happened…/(fg 5).*

The RNs discussed that the Senior Alerts content was valuable for educating NAs as well as RNs students. The RNs experienced that following the process in accordance with Senior Alert improved the likelihood for older persons to obtain equal risk assessments and adequate care interventions. Senior Alert was described as being particularly important when the older persons could not express themselves, and it enabled health care staff to risk assess in similar ways. An RN explained: *…./And that is why I am very glad about this Senior Alert because it makes us focus on the same thing, we are forced to do it and not feel many things (fg 4).* Although using Senior Alert was considered a good strategy, some RNs established their own strategies in preventive work.

The managers discussed that health care staff always worked preventively; however, previously, this work was not systematically or structured performed. The managers described that finding strategies helped workers, particularly NAs, obtain an overview of the entire preventive process. One manager expressed it as follows: *I feel that there are many hearts in this (preventive work). So, it is just a matter of finding good structures and getting opportunities to do it structured together and share knowledge with each other (fg 1).*

## Discussion

The current study reports a commitment and responsibility among NAs, RNs and managers in nursing homes considering the prevention of pressure ulcers, malnutrition, poor oral health and falls in general and according to the Senior Alert care process, yet, this work had to be done in a complex environment. Nevertheless, for preventive work to be successful, team effort and finding useful strategies are necessary.

NAs, RNs, managers and older persons in nursing homes relate to each other and the environment in nonlinear ways; therefore, we argue that the prevention of pressure ulcers, malnutrition, poor oral health and falls among older persons in nursing homes is not a straightforward process. In contrast, in this work, each situation is unique, and many factors and challenges, both at the individual and organizational levels, are involved. C. Lannering et al. (2017) stressed that organizational factors hinder preventive work and are concordant with our findings. In the current study, it is clear that the different professionals struggled with challenges at different levels, i.e., the micro, meso and macro levels. While the micro level refers to patients, the meso level concerns organizational culture and climate, willingness to change, support and structures. The macro level, on the other hand, concerns the wider environment, including policies, guidelines, evidence, regulations and legislation (Nilsen & Bernhardsson, 2019).

Overall, the NAs in this study struggled with challenges on a micro level concerning older person. For instance, they experienced difficulties in preventing the risk for poor oral health and malnutrition, particularly among older persons with dementia diagnoses. It was difficult, on the one hand, to risk assessing oral health and, on the other hand, to execute care interventions regarding oral intake. Since poor oral health and malnutrition are associated (Van Lancker et al., 2012) and decreased cognitive function is, in turn, associated with increased risk for malnutrition (Johansson et al., 2017), this may explain why the NAs in the current study particularly voiced their concern in preventing the risk for poor oral health and malnutrition among older persons with dementia diagnosis. Because demographic aging is occurring worldwide, more people are expected to develop dementia (Winblad et al., 2016); our results illuminate the need for increased knowledge among NAs working with older persons with dementia diagnosis in nursing homes and are in line with a recent systematic review (Li et al., 2021). In addition, a recent report from the Swedish National Board of Health and Welfare (2021) revealed that older persons in nursing homes have reduced or no access to mobile oral home care because of the national restrictions put in place due to COVID-19. In the same report, terrifyingly, older persons were not risk assessed for the risk for poor oral health at the same level, thus pointing towards the need for increased knowledge among health care staff in how to prevent the risk for poor oral health regardless of receiving support from mobile oral home care. Bellander et al. (2021) and Hoben et al. (2017) also reported the importance of educating nursing staff to prevent or maintain good oral health in older persons.

The RNs and managers, on the other hand, experienced challenges at the meso and macro levels. For instance, because the nursing record and Senior Alert were not compatible, this led to duplicated documentation and was time-consuming. This could be attributed to challenges at both the meso and macro levels since it concerns the organizational structure, as well as regulations at a higher level. Another challenge at the macro level was that NAs and RNs work under different legislations and document their work in different record systems, which, in turn, impedes everyday work.

The NAs in our study have a secondary education in nursing and a formal education regarding the preventive work process, as outlined by Senior Alert. However, the lack of knowledge among NAs regarding the preventive work was recognized as challenging among all professionals in the current study, including themselves. The lack of knowledge among the NAs aligns with a previous study (Dalvand et al., 2018) and could be seen as an organizational challenge at the macro level. Older persons in nursing homes today have a need for increased nursing competence, but worryingly, the need for increased nursing competence has not been adjusted (Knutsen Glette et al., 2018). In fact, among those employed in Swedish municipalities, only 60% have been educated in nursing care (Swedish National Board of Health and Welfare, 2020). Moreover, because a high level of staff turnover is recognized as challenging in the current study, this might jeopardize competence in nursing homes and pose a serious threat to the quality of care in elderly care (Zúñiga et al., 2015). Additionally, because the RNs in our study were concerned about NAs not fully understanding the terminology in Senior Alert and since it was an unclear division of labor between the RNs and managers who were responsible it actually was to educate the NAs, there is a need for increased knowledge among them. Indeed, all the professionals expressed a need for increased knowledge to maintain and increase competence levels among those working in nursing homes.

Although managers must give NAs the opportunity to learn, and they need to understand that preventive work is time-consuming, our study adds new knowledge by exploring whether people with a leading position realize the benefits of and support using a preventive work process, as outlined by Senior Alert. Similar to a previous study (Skytt et al., 2016), the participants in this study were aware of the existing challenges and, importantly, positive towards being given opportunities to improve preventive work and, as a result, enhance the quality of care among older persons in nursing homes.

In the current study, the prevention of these health risks among older persons in nursing homes unquestionably required a team effort. This part of our findings aligns with a recent study by (Westerlund et al., 2021), which pointed towards the importance of teamwork in preventive work, and a study by (Lavallée et al., 2018), which reported that teamwork within and between nursing home staff facilitates this work. Of course, NAs, being the main providers of care and close to the older person, are key persons in the team; however, the RN role on the team is crucial. This is in line with a Danish study that stresses the importance of the RN role in, for instance, preventing pressure ulcers among older patients (Lindhardt et al., 2020), and a recent systematic review found that RNs were consistently associated with a reduction in the prevalence of pressure ulcers (Clemens et al., 2020). In the current study, however, the physician did not seem to have a significant role on the team. This discrepancy needs to be further explored. Committed leadership, on the other hand, was important when working preventively, which indicates, similar to previous research (Seljemo et al., 2020), that managers have a key role in the team and, importantly, can create prerequisites for NAs and RNs to perform preventive work. Consequently, a multidisciplinary team is needed to prevent pressure ulcers, malnutrition, poor oral health and falls among older persons in nursing homes.

Impressively, although preventive work had to be done in a complex environment, and all the participants struggled with challenges, they found strategies that facilitated this work. Following a preventive work process, as outlined by Senior Alert, was a strategy that facilitated this work, thus substantiating the study by (Gusdal et al., 2021) and emphasizing the importance of a comprehensive preventive work process in this environment. However, although it is important to find strategies that work in everyday work, one of the main challenges seems to lie in the variety of knowledge found among those working in nursing homes, particularly among NAs. This variety suggests that there is an urgent need for a pedagogical educational intervention that can ease preventive work for NAs. Disseminating knowledge in a systematic way in nursing homes that is sustainable is difficult (Rantz et al., 2010); however, since complex interventions may work best if they are tailored to the local context (Nilsen & Bernhardsson, 2019), the potential value of this qualitative study, before developing and testing an intervention, is likely to enhance an effective intervention (O'Cathain et al., 2013).

### 6.1 Limitations

It is important to acknowledge the limitations of this study. First, even though the focus group interviews lasted between 63 and 106 min, the discussions in the focus groups might have been affected since they were digital. Nevertheless, the discussions were energetic and indicated that there were no barriers to the respondents sharing experiences. Second, there is always a possibility that the participants withheld vital information to avoid voicing critical views; however, to avoid power relations, homogeneous focus group interviews were conducted. In addition, the moderator (MN) tried to create a respectful and safe atmosphere. Another possible limitation is that the NAs in this study had additional education regarding the preventive work process, as outlined by Senior Alert. This does not reflect all NAs working in nursing homes in southern Sweden and can be attributed to the question of respondent bias. Another limitation could be that only four managers participated in this study. Although our study was conducted in southern Sweden and linked to a particular context, the prevention of pressure ulcers, malnutrition, poor oral health and falls among older persons should be a priority in all nursing homes; hence, the knowledge gained from this study might be valuable for all those working in nursing homes and may be transferable to similar contexts.

## Conclusion

There is a commitment and responsibility among NAs, RNs and managers regarding preventive work. Clearly, making a team effort and finding useful strategies are necessary for this work to be successful. However, based on our findings, it is plausible to claim that the prevention of pressure ulcers, malnutrition, poor oral health and falls among older persons in nursing homes is complex. Challenges, both at the individual and organizational levels, are involved, which implies that smoother organizational routines facilitating this preventive work are needed. Although NAs, RNs and managers are good at finding strategies that facilitate preventive work, one of the main challenges seems to lie in the variety of knowledge among those working in nursing homes, particularly among NAs. All the professionals voiced a need for an increased level of knowledge, particularly among NAs. Hence, to better equip those working in nursing homes, especially NAs, we suggest a tailored pedagogical educational intervention aimed at increasing the level of knowledge concerning the entire preventive process regarding pressure ulcers, malnutrition, poor oral health and falls among older persons in nursing homes.

## Conflict of Interest

None.

## Funding sources

No external funding.

## Declaration of Competing Interest

None.
